# Long-term cancer risk in women given diethylstilbestrol (DES) during pregnancy

**DOI:** 10.1054/bjoc.2000.1521

**Published:** 2001-01-01

**Authors:** L Titus-Ernstoff, E E Hatch, R N Hoover, J Palmer, E R Greenberg, W Ricker, R Kaufman, K Noller, A L Herbst, T Colton, P Hartge

**Affiliations:** Department of Community and Family Medicine, Dartmouth Medical School and the Norris Cotton Cancer Center, Lebanon, NH 03756; Division of Cancer Epidemiology and Genetics, National Cancer Institute, Bethesda, MD 20892; Slone Epidemiology Unit, Boston University School of Public Health, Brookline, MA 02446-4955; Department of Medicine, Dartmouth Medical School, Lebanon, NH 03756; Information Management Services, Rockville, MD 20852; Department of Obstetrics and Gynecology, Baylor College of Medicine, Houston, TX 77030; Department of Obstetrics and Gynecology, New England Medical Center, Boston, MA 02111; Department of Obstetrics and Gynecology, University of Chicago, Chicago, IL 60637; Department of Epidemiology and Biostatistics, Boston University School of Public Health, Boston, MA 02118, USA

**Keywords:** DES, oestrogens, breast cancer, endometrial cancer, ovarian cancer, cancer

## Abstract

From 1940 through the 1960s, diethylstilbestrol (DES), a synthetic oestrogen, was given to pregnant women to prevent pregnancy complications and losses. Subsequent studies showed increased risks of reproductive tract abnormalities, particularly vaginal adenocarcinoma, in exposed daughters. An increased risk of breast cancer in the DES-exposed mothers was also found in some studies. In this report, we present further follow-up and a combined analysis of two cohorts of women who were exposed to DES during pregnancy. The purpose of our study was to evaluate maternal DES exposure in relation to risk of cancer, particularly tumours with a hormonal aetiology. DES exposure status was determined by a review of medical records of the Mothers Study cohort or clinical trial records of the Dieckmann Study. Poisson regression analyses were used to estimate relative risks (RR) and 95% confidence intervals (CI) for the relationship between DES and cancer occurrence. The study results demonstrated a modest association between DES exposure and breast cancer risk, RR = 1.27 (95% CI = 1.07–1.52). The increased risk was not exacerbated by a family history of breast cancer, or by use of oral contraceptives or hormone replacement therapy. We found no evidence that DES was associated with risk of ovarian, endometrial or other cancer. © 2001 Cancer Research Campaign http://www.bjcancer.com


British Journal of Cancer (2001) **84**(1), 126–133
© 2001 Cancer Research Campaign
doi: 10.1054/ bjoc.2000.1521, available online at http://www.idealibrary.com on IDEAL®

http://www.bjcancer.com

### Long-term cancer risk in women given diethylstilbestrol (DES) during pregnancy

**L Titus-Ernstoff[1], EE Hatch[2], RN Hoover[2], J Palmer[3], ER Greenberg[1,4], W Ricker[5], R Kaufman[6], K Noller[7], AL Herbst[8], T Colton[9] and P Hartge[3]**

[1]Department of Community and Family Medicine, Dartmouth Medical School, and the Norris Cotton Cancer Center, Lebanon, NH 03756; [2]Division of Cancer Epidemiology and Genetics, National Cancer Institute, Bethesda, MD 20892; [3] Slone Epidemiology Unit, Boston University School of Public Health, Brookline, MA 02446-4955; [4]Department of Medicine, Dartmouth Medical School, Lebanon, NH 03756; [5]Information Management Services, Rockville, MD 20852; [6]Department of Obstetrics and Gynecology, Baylor College of Medicine, Houston, TX 77030; [7]Department of Obstetrics and Gynecology, New England Medical Center, Boston, MA 02111; [8]Department of Obstetrics and Gynecology, University of Chicago, Chicago, IL 60637; [9]Department of Epidemiology and Biostatistics, Boston University School of Public Health, Boston, MA 02118, USA

**Summary** From 1940 through the 1960s, diethylstilbestrol (DES), a synthetic oestrogen, was given to pregnant women to prevent pregnancy complications and losses. Subsequent studies showed increased risks of reproductive tract abnormalities, particularly vaginal adenocarcinoma, in exposed daughters. An increased risk of breast cancer in the DES-exposed mothers was also found in some studies. In this report, we present further follow-up and a combined analysis of two cohorts of women who were exposed to DES during pregnancy. The purpose of our study was to evaluate maternal DES exposure in relation to risk of cancer, particularly tumours with a hormonal aetiology. DES exposure status was determined by a review of medical records of the Mothers Study cohort or clinical trial records of the Dieckmann Study. Poisson regression analyses were used to estimate relative risks (RR) and 95% confidence intervals (CI) for the relationship between DES and cancer occurrence. The study results demonstrated a modest association between DES exposure and breast cancer risk, RR = 1.27 (95% CI = 1.07–1.52). The increased risk was not exacerbated by a family history of breast cancer, or by use of oral contraceptives or hormone replacement therapy. We found no evidence that DES was associated with risk of ovarian, endometrial or other cancer. © 2001 Cancer Research Campaign http://www.bjcancer.com

**Keywords**: DES; oestrogens; breast cancer; endometrial cancer; ovarian cancer; cancer

Diethylstilbestrol (DES) is a synthetic estrogen that was used widely from about 1940 through the 1960s to prevent late complications of pregnancy. Although estimates vary, two million women in the US (Noller and Fish, 1974), and perhaps four million women worldwide (Newbold, 1993) have been exposed to DES during pregnancy. In 1953, the results of a clinical trial conducted at the University of Chicago failed to demonstrate that DES was beneficial for preventing pregnancy complications (Dieckmann et al, 1953). Nevertheless, DES remained in use for this indication until the early 1970s, when adverse effects, including clear cell adenocarcinoma of the vagina, were noted in prenatally exposed daughters (Herbst and Scully, 1971).

Several studies have examined breast cancer risk among women who received DES during pregnancy. Most found an increase of risk (Bibbo et al, 1978; Beral and Colwell, 1980; Vessey et al, 1983; Greenberg et al, 1984; Hadjimichael et al, 1984; Colton et al, 1993) or mortality (Calle et al, 1996), although the association was not always statistically significant (Bibbo et al, 1978; Beral and Colwell, 1980; Vessey et al, 1983; Hadjimichael et al, 1984). A few earlier studies also suggested elevated risks of endometrial (Hoover et al, 1976; Autunes et al, 1979) and ovarian cancer (Hoover et al, 1977; Bibbo et al, 1978; Hadjimichael et al, 1984) among women who took DES. A current concern is whether the use of hormone replacement therapy (HRT) may further increase the risk of breast cancer in DES-exposed women.

*Received 19 July 2000*
*Revised 29 August 2000*
*Accepted 30 August 2000*

*Correspondence to:* L Titus-Ernstoff

### METHODS

The purpose of this study was to evaluate long-term cancer risk, particularly breast cancer risk, among women who were exposed to DES during pregnancy. In this report, we present further follow-up and a combined analysis of cancer risk in two cohorts of DES-exposed women, the Mothers Study cohort, and the Dieckmann Study cohort. The Mothers Study comprised women ascertained in the early 1980s through a review of medical records at the Mayo Clinic in Rochester, MN, Mary Hitchcock Memorial Hospital (MHMH) in Hanover, NH, a high-risk pregnancy clinic at the Boston Lying-In Hospital (BLI), in Boston, MA, and a private obstetrics practice in Portland, ME (Greenberg et al, 1984; Colton et al, 1993). At MHMH, the private obstetrics practice in Portland and the Mayo Clinic cohort members were identified through a retrospective review of obstetrics records for the period 1940–1960. DES-exposed women were those whose records indicated that DES (or, rarely, another nonsteroidal oestrogen) had been prescribed during at least one pregnancy resulting in a live birth. The date of the first DES-exposed live birth was the study entry date. Records from the same four sources were used to assemble a comparison sample of unexposed women who had delivered at least one live birth during the same time period (1940–1960), and whose charts did not indicate exogenous oestrogen use during any of their pregnancies. Unexposed women were matched within ± 2 years to the DES-exposed women's birth dates, and were assigned the same date of study entry as the exposed woman to whom they were matched. Comparable ascertainment procedures were followed at BLI, except that exposed women were identified through a high-risk pregnancy clinic, and unexposed women were sampled from the card file of all patients. Previous follow-up of women in the DES Mothers Study cohort occurred in 1981, 1986, and 1989 (Greenberg et al, 1984; Colton et al, 1993).

The Dieckmann Study cohort consists of women participating in a clinical trial that examined the effects of DES on pregnancy outcomes. The Dieckmann trial, conducted in the early 1950s at the University of Chicago, enrolled women who were 6–20 weeks pregnant. Women who entered the prenatal clinic were alternately assigned to receive DES or placebo; the date of pregnancy outcome was the study entry date. Participants in the Dieckmann Study were evaluated in 1976 for cancer outcomes (Bibbo et al, 1978). In 1992, members of the cohort were re-contacted in conjunction with renewed follow-up of the Mothers Study cohort.

### Follow-up

During 1992, we undertook intensive tracing efforts to locate women who had been previously followed. A total of 625 women (262 exposed, 363 unexposed), comprising 8% of the initial cohorts (7% of exposed, 9% of unexposed), could not be located (Table 1). In 1994, we sent follow-up questionnaires to women who were presumed alive, who had not previously refused further contact, and for whom addresses were available. If a woman did not respond after two mailings, we attempted a telephone interview. Through these procedures, we obtained completed questionnaires from 4836 women approached for follow-up, including 2434 (88%) exposed and 2402 (89%) unexposed women. An additional 638 women (327 exposed, 311 unexposed) either refused to participate or did not respond to our efforts to contact them. We searched the National Death Index for women who could not be located, and obtained death certificates for women known to have died. Death certificates were coded by a nosologist. A total of 1659 deaths (856 exposed, 803 unexposed) were ascertained since the initiation of follow-up. Complete follow-up (either a completed 1994 questionnaire or a death certificate) was obtained for 84% (6495/7758) of women (85% exposed, 83% unexposed) who had been enrolled in the initial cohorts. Follow-up was more complete for the Mothers Study cohort (88%) than the Dieckmann Study cohort (69%), and was comparable for exposed and unexposed women in each cohort.

Cancers recorded prior to the 1994 follow-up had been confirmed by the medical record or death certificate. A total of 621 new invasive cancers were reported on the 1994 questionnaire; medical records were obtained for 551 (89%), of which 510 (93%) confirmed the reported diagnosis. The reported cancers included 251 invasive breast cancers; medical records were obtained for 231 (92%), of which 225 (97%) confirmed the reported diagnosis.

### Statistical analyses

The analyses presented here are based on the diagnosis of invasive cancer confirmed by a review of the medical record, or ascertained through underlying cause of death listed on the death certificate. We compared cancer risk in the DES-exposed women to that in the unexposed cohort and in the general population. Person-years were calculated from the date of study entry until the earliest of the following events: date of cancer diagnosis, date of death, or the date of the last known follow-up. If a woman had more than one cancer diagnosis, person-years were accrued until the date of the diagnosis of interest; in the analyses of all cancers, person-years were accrued until the earliest cancer diagnosis. A total of 3844 exposed women (contributing 143 567 person years) and 3716 unexposed women (contributing 139 735 person years) were available for the combined cancer analyses. For analyses of endometrial and ovarian cancer, women who had hysterectomies exited the analyses at the date of surgery.

Analyses were conducted separately for the Mothers Study and Dieckmann Study cohorts, and in the two cohorts combined. Poisson regression analyses were used to estimate the risk of cancer in DES-exposed vs unexposed women, controlling for age, calendar year, and cohort (in the combined analyses) (Breslow and Day, 1987). For breast cancer, we evaluated potential confounding by years of education, family history of breast cancer, age at menarche, oral contraceptive use, number of pregnancy losses, age at first full-term birth, parity, menopausal status and HRT use. Potential confounders in analyses of ovarian cancer risk were oral contraceptive use, parity, and use of HRT; potential confounders in analyses of endometrial cancer risk were body mass index, oral contraceptive use, parity, and HRT. In all analyses, menopause and HRT were treated as time-dependent variables. Other variables were evaluated using the categories shown in Table 2. In general, covariates were based on the most recent information, although age at menarche was taken from the earliest record available.

We used stratified analyses to evaluate the effect of DES on breast cancer risk according to age at study entry, attained age, and time since exposure. The influence of DES was also evaluated according to family history of breast cancer, oral contraceptive use, number of pregnancy losses, age at first full-term birth, parity,

**Table 1** Status of cohorts

| | Mothers Study | | Dieckmann Study | | |
|---|---|---|---|---|---|
| | Exposed | Unexposed | Exposed | Unexposed | Total |
| Initial members | 3053 | 3075 | 826 | 804 | 7758 |
| Ever followed | 2885 | 2816 | 693 | 668 | 7062 |
| Deceased | 698 | 678 | 158 | 125 | 1659 |
| Approached in 1994 | 2243 | 2215 | 518 | 498 | 5474 |
| Questionnaire returned | 2019 | 1978 | 415 | 424 | 4836 |
| Questionnaire not returned | 224 | 237 | 103 | 74 | 638 |
| Whereabouts unknown | 112 | 182 | 150 | 181 | 625 |

*© 2001 Cancer Research Campaign*

**Table 2** Number (and percent) of DES-exposed and- unexposed women according to select characteristics, by cohort

| | Mothers Study | | Dieckmann Study | |
|---|---|---|---|---|
| **Factor** | **Exposed** | **Unexposed** | **Exposed** | **Unexposed** |
| Age at study entry (years) | | | | |
| < 25 | 882 (29.1) | 868 (28.8) | 218 (26.4) | 219 (27.2) |
| 25–29 | 1059 (35.0) | 1051 (34.9) | 302 (36.6) | 293 (36.4) |
| 30–34 | 643 (21.2) | 669 (22.2) | 189 (22.9) | 196 (24.4) |
| ≥ 35 | 445 (14.7) | 424 (14.1) | 117 (14.2) | 96 (11.9) |
| Body mass index[a] (kg m$^{-2}$) | | | | |
| < 21 | 319 (13.8) | 297 (13.4) | 46 (9.6) | 60 (12.1) |
| 21–23 | 900 (38.9) | 813 (36.6) | 140 (29.3) | 122 (24.6) |
| 24–27 | 741 (32.1) | 776 (34.9) | 151 (31.6) | 181 (36.5) |
| ≥ 28 | 351 (15.2) | 336 (15.1) | 141 (29.5) | 133 (26.8) |
| missing | 718 | 790 | 348 | 308 |
| Education (years)[b] | | | | |
| 0–8 | 164 (6.7) | 188 (8.0) | – | – |
| 9–12 | 1205 (49.0) | 1139 (48.5) | – | – |
| 13–16 | 899 (36.5) | 838 (35.7) | – | – |
| ≥ 17 | 193 (7.8) | 182 (7.8) | – | – |
| missing | 568 | 665 | 826 | 804 |
| Family history of breast cancer | | | | |
| no | 2606 (86.0) | 2660 (88.3) | 729 (88.3) | 703 (87.4) |
| yes | 423 (14.0) | 352 (11.7) | 97 (11.7) | 101 (12.6) |
| Age at menarche | | | | |
| < 12 | 404 (14.6) | 322 (12.3) | 120 (15.0) | 126 (16.4) |
| 12, 13 | 1524 (54.9) | 1492 (56.9) | 446 (55.6) | 413 (53.8) |
| ≥ 14 | 848 (30.6) | 808 (30.8) | 236 (29.4) | 228 (29.7) |
| missing | 253 | 390 | 24 | 37 |
| Oral contraceptive use | | | | |
| no | 1923 (78.8) | 1872 (80.3) | 578 (79.3) | 564 (82.1) |
| yes | 518 (21.2) | 460 (19.7) | 151 (20.7) | 123 (17.9) |
| missing | 588 | 680 | 97 | 117 |
| Pregnancy losses | | | | |
| 0 | 1038 (39.2) | 1558 (63.3) | 511 (66.2) | 482 (66.0) |
| 1 | 764 (28.9) | 616 (25.0) | 165 (21.4) | 158 (21.6) |
| ≥ 2 | 846 (32.0) | 288 (11.7) | 96 (12.4) | 90 (12.3) |
| missing | 381 | 550 | 54 | 74 |
| Age at first full-term birth | | | | |
| < 20 | 367 (12.2) | 441 (14.8) | 69 (8.5) | 75 (9.5) |
| 20–24 | 1329 (44.2) | 1501 (50.2) | 345 (42.3) | 328 (41.6) |
| 25–29 | 895 (29.8) | 772 (25.8) | 289 (35.4) | 280 (35.5) |
| ≥ 30 | 414 (13.8) | 275 (9.2) | 113 (13.9) | 105 (13.3) |
| missing | 24 | 23 | 10 | 16 |
| Parity | | | | |
| 1,2 | 589 (24.2) | 591 (25.5) | 257 (32.5) | 241 (32.0) |
| 3,4 | 1147 (47.2) | 1113 (48.0) | 392 (49.6) | 363 (48.2) |
| ≥ 5 | 694 (28.6) | 613 (26.5) | 141 (17.9) | 149 (19.8) |
| missing | 599 | 695 | 36 | 51 |
| Age at menopause | | | | |
| < 39 | 321 (13.8) | 265 (11.8) | 44 (7.8) | 51 (9.1) |
| 40–44 | 552 (23.8) | 474 (21.2) | 77 (13.7) | 83 (14.9) |
| 45–49 | 532 (22.9) | 533 (23.8) | 160 (28.5) | 158 (28.3) |
| 50–54 | 756 (32.5) | 799 (35.7) | 218 (38.8) | 212 (37.9) |
| ≥ 55 | 163 (7.0) | 169 (7.5) | 63 (11.2) | 55 (9.8) |
| missing | 705 | 772 | 264 | 245 |
| Hormone replacement therapy | | | | |
| no | 1644 (65.4) | 1689 (70.0) | 536 (68.9) | 453 (62.5) |
| yes | 870 (34.6) | 725 (30.0) | 242 (31.1) | 272 (37.5) |
| missing | 515 | 598 | 48 | 79 |
| Hysterectomy | | | | |
| no | 1885 (62.2) | 2040 (67.7) | 548 (66.3) | 517 (64.3) |
| yes | 1144 (37.8) | 972 (32.3) | 278 (33.7) | 287 (35.7) |
| Ever smoked | | | | |
| no | 1187 (45.9) | 1158 (47.0) | 512 (62.2) | 507 (63.5) |
| yes | 1401 (54.1) | 1308 (53.0) | 311 (37.8) | 292 (36.6) |
| missing | 441 | 546 | 3 | 5 |

[a]BMI based on weight at age 50 for DES Mothers Study cohort, and on current weight at most recent questionnaire response for the Dieckmann cohort; [b]not available for the Dieckmann cohort.

 *© 2001 Cancer Research Campaign*

menopausal status, and HRT use. Additional analyses assessed the influence of DES on ovarian and endometrial cancer risk according to use of HRT. Potential interactions between DES and other risk factors were assessed using the likelihood ratio test (Breslow and Day, 1987).

Age-standardized cancer rates for DES-exposed and unexposed women were calculated using the 1970 US population as a standard. For external comparisons, we used cancer incidence rates for women from the Connecticut Tumor Registry (Heston et al, 1986) for the years prior to 1970, and from the Surveillance, Epidemiology, and End Results (SEER) program (Ries et al, 1999) for the years 1970–1994 (for white women). Expected numbers of cancers were generated by applying age- and calendar year-specific incidence rates to the appropriate person-years at risk. We computed standardized incidence ratios (SIRs) and their 95% confidence intervals assuming a Poisson distribution for the observed numbers of cancers (Breslow and Day, 1987).

For exposed women, we calculated the proportion of breast cancer due to DES exposure (i.e., the attributable risk) as RR–1/RR (Kelsey et al, 1986).

### RESULTS

Compared to the Dieckmann Study, Mothers Study participants had higher parity, younger age at first full-term birth, younger age at menopause, and higher frequency of cigarette smoking (Table 2). Mothers Study participants also appeared to have lower body mass index, but this likely reflected the earlier age at which weight was ascertained in this cohort (average 50 years), compared to the Dieckmann Study cohort (average 70 years): They also reported more pregnancy losses, the primary clinical indication for DES use.

Within each of the cohorts, exposed and unexposed women were generally comparable when considered according to education (available only in the Mothers Study), age at menarche, parity, and smoking histories (Table 2). In the Mothers Study cohort, exposed women were older than unexposed women at first full-term birth, reported a higher number of pregnancy losses, and were more likely to have a family history of breast cancer.

In comparison to unexposed women, DES-exposed women in both cohorts had a slightly elevated cancer risk, although the effect was small in the Mothers Study, and consistent with chance in both cohorts (Table 3). In the combined cohort, the RR of cancer was 1.10 (95% CI = 0.99–1.23). The only significantly elevated risk was for breast cancer, which accounted for most of the excess observed in all cancer. Although risks of lung cancer and non-Hodgkins lymphomas were somewhat elevated in both cohorts, these were not statistically significant. The age-standardized rates for all cancer per 100 000 were 289.2 for exposed women, and 249.6 for unexposed women. Relative to the general US population rates, cancer rates for the combined cohort were reduced for DES-exposed women (SIR = 0.88, 95% CI = 0.82–0.95), and for unexposed women (SIR = 0.80, 95% CI = 0.74–0.87). The SIRs were similar when based on self-reported cancers.

DES exposure was associated with an increased breast cancer risk both in the Mothers Study cohort (RR = 1.29, 95% CI = 1.06–1.57) and, though non-significantly, the Dieckmann Study (RR = 1.23, 95% CI = 0.85–1.78) (Table 3). When unconfirmed cases were included in the analyses, the results were similar for the Mothers Study (RR = 1.26, 95% CI = 1.04–1.53) and Dieckmann Study cohorts (RR = 1.26, 95% CI = 0.88–1.82). For the combined cohort, the relative risk (RR) of breast cancer was 1.27 (95% CI = 1.07–1.52); the association was essentially unchanged (RR = 1.25, 95% CI = 1.05–1.50) after adjustment for potential confounders.

**Table 3** Number of confirmed cancers[a], relative risks[b] (RR) and 95% confidence intervals (CI) for cancer sites, and for all cancers, by cohort

| | Mothers Study | | | Dieckmann Study | | | Combined cohort |
|---|---|---|---|---|---|---|---|
| **Cancer site** | **Exp.** | **Unexp.** | **RR (95% CI)** | **Exp.** | **Unexp.** | **RR (95% CI)** | **RR (95% CI)** |
| All cancers | 561 | 509 | 1.07 (0.95–1.21) | 132 | 100 | 1.28 (0.98–1.66) | 1.10 (0.99–1.23) |
| Oesophagus | 2 | 6 | 0.32 (0.07–1.06) | 0 | 0 | – | – |
| Stomach | 9 | 5 | 1.74 (0.58–5.20) | 2 | 0 | – | 2.13 (0.74–6.14) |
| Colorectal | 71 | 71 | 1.06 (0.77–1.47) | 13 | 7 | 1.80 (0.72–4.51) | 1.13 (0.83–1.53) |
| Liver | 6 | 5 | 1.16 (0.35–3.79) | 1 | 0 | – | 1.35 (0.43–4.25) |
| Biliary | 5 | 3 | 1.67 (0.40–6.99) | 0 | 0 | – | – |
| Pancreas | 18 | 16 | 1.09 (0.55–2.13) | 1 | 1 | 0.97 (0.06–15.55) | 1.08 (0.56–2.08) |
| Lung | 72 | 55 | 1.27 (0.89–1.80) | 16 | 10 | 1.59 (0.72–3.50) | 1.31 (0.95–1.81) |
| Lymph/haemat[c] | 46 | 39 | 1.14 (0.74–1.75) | 5 | 4 | 1.22 (0.33–4.55) | 1.14 (0.76–1.72) |
| Non-Hodgkins | 31 | 21 | 1.43 (0.82–2.48) | 3 | 2 | 1.42 (0.24–8.49) | 1.43 (0.84–2.42) |
| Hodgkins | 1 | 4 | 0.24 (0.03–2.16) | 0 | 0 | – | 0.24 (0.03–2.16) |
| Melanoma | 15 | 16 | 0.90 (0.45–1.83) | 3 | 2 | 1.56 (0.26–9.38) | 0.97 (0.50–1.86) |
| Connective tissue | 7 | 3 | 2.30 (0.59–8.89) | 0 | 0 | – | – |
| Breast | 227 | 172 | 1.29 (1.06–1.57) | 63 | 49 | 1.23 (0.85–1.78) | 1.27 (1.07–1.52) |
| Cervical | 11 | 21 | 0.51 (0.25–1.06) | 5 | 1 | 4.71 (0.55–40.45) | 0.71 (0.37–1.35) |
| Endometrial | 34 | 35 | 1.01 (0.63–1.62) | 8 | 11 | 0.66 (0.27–1.65) | 0.92 (0.60–1.39) |
| Ovarian | 22 | 32 | 0.67 (0.39–1.15) | 6 | 6 | 0.94 (0.30–2.93) | 0.72 (0.44–1.17) |
| Urinary tract[d] | 8 | 9 | 0.85 (0.33–2.21) | 4 | 1 | 3.65 (0.41–32.73) | 1.15 (0.50–2.66) |
| Bladder | 10 | 7 | 1.37 (0.52–3.61) | 1 | 1 | 0.95 (0.06–15.22) | 1.32 (0.53–3.29) |
| Brain | 6 | 10 | 0.58 (0.21–1.59) | 1 | 2 | 0.49 (0.04–5.44) | 0.56 (0.22–1.42) |
| Thyroid | 7 | 8 | 0.85 (0.31–2.33) | 1 | 3 | 0.33 (0.03–3.15) | 0.71 (0.28–1.75) |
| Unspecified | 14 | 22 | 0.61 (0.31–1.20) | 4 | 4 | 0.95 (0.24–3.81) | 0.67 (0.37–1.22) |
| Person years | 115 853 | 112 840 | | 27 714 | 26 886 | | |

[a]Includes sites with at least eight confirmed cases; [b]adjusted for age, calendar year, and the interaction between age and calendar year; [c]lymphatic and haematopoietic malignancies, ICD-9 codes 200–208; [d]other than urinary bladder

*© 2001 Cancer Research Campaign*

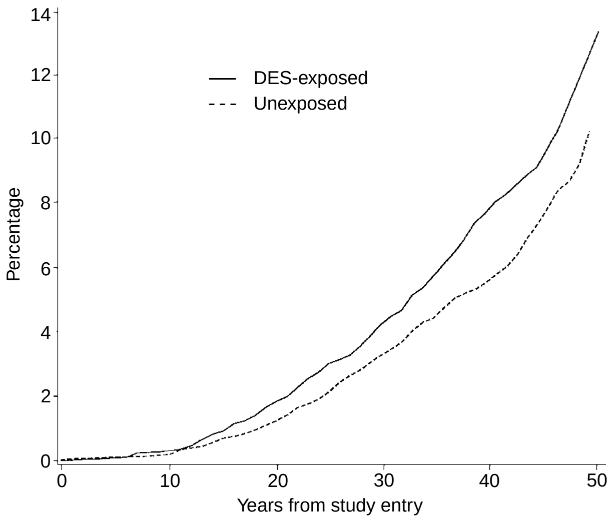

**Fig. 1**

The combined cohort results were also similar when study exit dates for nonrespondents and women lost to follow-up were extended to the end of the follow-up period. The graph of cumulative breast cancer incidence for the combined cohort, by exposure status, indicates that risk began to diverge within 10 years of exposure (Figure 1).

The age-standardized breast cancer rates per 100 000 were 106.9 for exposed women, and 83.9 for unexposed women in the combined cohort. Relative to the general US population, the incidence rate was slightly elevated among DES-exposed women (SIR = 1.10, 95% CI = 0.98–1.23), and slightly but significantly reduced among unexposed women (SIR = 0.86, 95% CI = 0.75–0.98). The SIRs were similar when based on self-reported breast cancers.

There was little indication that the association between DES and breast cancer risk differed according to age at study entry or attained age (Table 4). The increased risk was evident for four decades following DES exposure, but was not apparent 40 or more years after exposure. The association between DES and breast cancer risk was not significantly modified by other factors (Table 5) including family history of breast cancer, or oral contraceptive or HRT use. In an analysis confined to DES-exposed women, the RR for breast cancer associated with DES exposure during a first pregnancy compared to a subsequent pregnancy, was 1.15 (95% CI = 0.90–1.47).

In the combined cohort, DES exposure was not associated with increased risk of endometrial (RR = 0.92, 95% CI = 0.60–1.39) or ovarian cancer (RR = 0.72, 95% CI = 0.44–1.17) (Table 3); the results were similar when adjusted for potential confounders. The influence of DES on risk of ovarian cancer was not greater for women who used HRT (RR = 0.34, 95% CI = 0.09–1.28). The association between DES and risk of endometrial cancer was somewhat, but not significantly elevated for women who used HRT (RR = 1.19, 95% CI = 0.56–2.55). There was no evidence of a statistical interaction between DES and use of HRT in relation to either ovarian or endometrial cancer.

### DISCUSSION

Our results indicate that women who took DES during pregnancy have a modest increased of breast cancer but they showed no significant increase in risk of other cancers, including ovary or endometrium cancers. These results are based on the largest study to date of women with documented exposure to DES during pregnancy. Our analyses of the combined cohort were based on over 500 breast cancer cases, including an additional 225 new diagnoses ascertained since the previous follow-up. A unique feature of this study is the ability to compare the results of two cohorts, one identified through prenatal record review, and the other based on a clinical trial of the effectiveness of DES. This is

**Table 4** Relative risks[a] (RR) and 95% confidence intervals (CI) for the relation between DES exposure and breast cancer risk for the combined cohort, according to age at study entry, attained age, and time since DES exposure

| | DES-exposed | | Unexposed | | |
|---|---|---|---|---|---|
| **Factor** | ***n*** | **person years** | ***n*** | **person years** | **RR (95% CI)** |
| Age at study entry | | | | | |
| < 25 | 63 | 47 236 | 52 | 46 407 | 1.19 (0.82–1.71) |
| 25–29 | 102 | 50 602 | 85 | 49 986 | 1.20 (0.90–1.60) |
| 30–34 | 83 | 31 087 | 53 | 30 464 | 1.55 (1.09–2.18) |
| ≥ 35 | 42 | 17 338 | 31 | 15 696 | 1.21 (0.76–1.92) |
| Attained age[b] | | | | | |
| < 40 | 11 | 43 927 | 10 | 42 946 | 1.08 (0.46–2.55) |
| 40–49 | 55 | 35 799 | 36 | 34 863 | 1.49 (0.98–2.27) |
| 50–59 | 88 | 32 800 | 72 | 32 170 | 1.20 (0.88–1.63) |
| 60–69 | 96 | 24 544 | 71 | 23 912 | 1.32 (0.97–1.79) |
| ≥ 70 | 40 | 9092 | 32 | 8663 | 1.19 (0.75–1.89) |
| Time since exposure (years) | | | | | |
| 0–9 | 10 | 37 198 | 7 | 36 143 | 1.39 (0.53–3.64) |
| 10–19 | 53 | 36 119 | 36 | 35 184 | 1.44 (0.94–2.19) |
| 20–29 | 83 | 33 964 | 67 | 33 050 | 1.21 (0.87–1.66) |
| 30–39 | 101 | 27 975 | 65 | 27 840 | 1.52 (1.11–2.07) |
| ≥ 40 | 43 | 11 008 | 46 | 10 836 | 0.92 (0.61–1.39) |

[a]Adjusted for age, calendar year, the interaction between age and calendar year, and cohort; [b]Age at diagnosis for cases; age at exit for non-cases

 © 2001 Cancer Research Campaign

**Table 5** Relative risks[a] (RR) and 95% confidence intervals (CI), for the relationship between DES exposure and breast cancer risk in the combined cohort, according to select breast cancer risk factors

| | DES-exposed | | Unexposed | | | |
|---|---|---|---|---|---|---|
| **Factor** | ***n*** | **person years** | ***n*** | **person years** | **RR (95% CI)** | ***P***[c] |
| Family history of breast cancer | | | | | | |
| no | 227 | 125 544 | 178 | 124 333 | 1.25 (1.03–1.53) | 0.85 |
| yes | 63 | 20 719 | 43 | 18 220 | 1.31 (0.89–1.93) | |
| Oral contraceptive use | | | | | | |
| never | 188 | 102 691 | 141 | 101 125 | 1.31 (1.05–1.62) | 0.85 |
| ever | 47 | 26 968 | 36 | 23 790 | 1.16 (0.75–1.79) | |
| missing | 55 | 16 604 | 44 | 17 638 | | |
| Pregnancy losses | | | | | | |
| 0 | 126 | 62 185 | 118 | 83 530 | 1.43 (1.11–1.84) | 0.46 |
| ≥ 1 | 134 | 73 366 | 73 | 44 956 | 1.13 (0.85–1.50) | |
| missing | 30 | 10 706 | 30 | 14 063 | | |
| Age at first full-term birth (years) | | | | | | |
| < 20 | 20 | 16 347 | 21 | 18 155 | 1.14 (0.61–2.12) | 0.69 |
| 20–24 | 110 | 64 446 | 106 | 68 749 | 1.15 (0.88–1.50) | |
| 25–29 | 99 | 45 423 | 66 | 40 593 | 1.32 (0.97–1.81) | |
| ≥ 30 | 57 | 19 058 | 26 | 14 176 | 1.72 (1.07–2.75) | |
| missing | 4 | 989 | 2 | 879 | | |
| Parity | | | | | | |
| 1,2 | 65 | 33 142 | 52 | 33 054 | 1.26 (0.87–1.82) | 0.43 |
| 3,4 | 127 | 62 368 | 82 | 60 951 | 1.52 (1.15–2.00) | |
| ≥ 5 | 48 | 34 843 | 43 | 31 445 | 1.02 (0.68–1.55) | |
| missing | 50 | 15 910 | 44 | 17 103 | | |
| Menopausal status[b] | | | | | | |
| premenopause | 29 | 56 020 | 20 | 55 951 | 1.50 (0.85–2.65) | 0.23 |
| postmenopause | 179 | 67 321 | 151 | 64 122 | 1.14 (0.92–1.41) | |
| missing | 82 | 22 916 | 50 | 22 475 | | |
| Hormone replacement therapy[b] | | | | | | |
| no | 171 | 105 589 | 126 | 105 384 | 1.37 (1.08–1.72) | 0.64 |
| yes | 48 | 19 632 | 40 | 17 533 | 1.10 (0.72–1.68) | |
| missing | 71 | 21 036 | 55 | 19 630 | | |

[a]Adjusted for age, calendar year, the interaction between age and calendar year, and cohort; [b]Treated as a time-dependent variable; [c]*P* value for the interaction with DES, based on the likelihood ratio test.

also the first study with both confirmed DES exposure and reasonable statistical power to assess whether DES is associated with hormonally mediated cancers other than breast cancer.

Our finding of a 27% increased breast cancer risk is consistent with the earlier follow-up of the Mothers Study (Greenberg et al, 1984; Colton et al, 1993), and with studies of Connecticut women (Hadjimichael et al, 1984) and of fatal breast cancer (Calle et al, 1996). The initial report of the Dieckmann follow-up did not indicate an effect of DES on breast cancer risk, but a re-analysis of the data showed a non-significant 47% increase in risk (based on 53 cases), and a nearly three-fold and marginally significant increase in breast cancer mortality (based on 16 cases) (Clark and Portier, 1979). Other studies with inconclusive (Beral and Colwell, 1980) or null results (Vessey et al, 1983) were also small in size.

In our data, the breast cancer risk was increased in DES-exposed women relative both to unexposed women and to US population rates. The women in our study were parous, perhaps accounting for the lower rate of breast cancer among unexposed women, relative to the general population. We also noted reduced rates of cancer at all sites combined, relative to the general population. This may reflect the better health of parous women, and protective lifestyle factors among women who, for the most part, sought obstetric care at teaching hospitals (Greenberg et al, 1984).

In this study, the influence of DES on breast cancer risk was fairly constant in the presence of other hormonal factors, including oral contraceptives, menopausal status, and HRT. Similar results were found in the study of fatal breast cancer (Calle et al, 1996). As in the initial follow-up of the Mothers Study cohort (Greenberg et al, 1984) and the study of fatal breast cancer (Calle et al, 1996), we found no evidence that the effect of DES was greater among women who had previous pregnancy losses or breast cancer in their families. We also found no evidence, in an analysis confined to DES-exposed women, that breast cancer risk was greater for women exposed during their first pregnancy, relative to a subsequent pregnancy.

In practice, women who were prescribed DES were often those with a history of high-risk pregnancies, raising the possibility that the relationship between DES and breast cancer risk was confounded by hormonal factors associated with pregnancy complications. In this study, we found no evidence of confounding by a history of pregnancy losses, or known breast cancer risk factors. The increased risk was also observed in the Dieckmann Study participants, whose use of DES was unrelated to potential confounders, including prior pregnancy losses. The association between DES and breast cancer risk is also unlikely to be an artifact of increased surveillance of the DES-exposed women. Previous follow-up of the Mothers Study cohort showed that the frequency of breast self-examination, breast examination by a physician, mammography, and stage of diagnosis were similar for exposed and unexposed women (Greenberg et al, 1984; Colton et al, 1993). In addition, previous follow-up of the Mothers (Colton et al, 1993) and Dieckmann Study cohorts (Bibbo et al,

*© 2001 Cancer Research Campaign* 

1978), and the results of a recent study (Calle et al, 1996) have shown an association between DES and breast cancer mortality. A possible limitation of our study was the long interim between evaluations of the Dieckmann cohort and consequent losses to follow-up. Ascertainment of vital status was comparable, however, for exposed and unexposed women in the Dieckmann cohort. We also found similar results with regard to breast cancer risk when the follow-up of untraceable and non-responding women was extended to the study end-date.

Although the increased risk associated with DES exposure is modest, an approximately 30% increased risk translates into a substantial number of excess cases for a disease as common as breast cancer. If DES is causally related to breast cancer risk, the results of our study indicate that it accounts for 21% of the breast cancer cases among DES-exposed women. A causal role would be more credible with evidence of a dose–response relationship, but this could not be evaluated in our study. Details of DES doses were often missing from the obstetrics records of Mothers Study participants, and were administered according to a standard protocol in the Dieckmann Study participants. However, in the study of Connecticut women, the increased breast cancer risk was similar for varying DES dose levels (Hadjimichael et al, 1984). The influence of DES exposure on breast cancer risk in cohorts with different average (or median) dose levels is not consistent with a dose–response relationship. For example, RRs were comparable in the study of Connecticut women, whose average total dose of DES was 2.1 g (Hadjimichael et al, 1984), and in the Dieckmann Study cohort (Clark and Portier, 1979), where the total dose was 11–12 g (Bibbo et al, 1978). A possible causal role for DES receives some support from evidence for an association between HRT and breast cancer risk (Colditz et al, 1995; CGHFBC, 1997; Schairer et al, 2000), generally with recent, long-term use; in contrast, DES exposure was brief, even for women with multiple exposed pregnancies, and the effect persisted for four decades. The dose levels of DES were relatively high, perhaps increasing the plausibility of an association with breast cancer risk. For women in the Dieckmann Study, for example, DES doses were 300-fold greater than the amount typically used for 1 year of hormone replacement (0.1 mg per day) (Noller and Fish, 1974). The persisting effect of DES may be due to the high dose levels administered, the pharmacological properties of non-steroidal oestrogens (DES) (which differ from those of natural conjugated oestrogens), or the susceptibility of breast tissue during pregnancy.

In this study, DES was not significantly associated with specific cancer sites other than the breast. In particular, DES was not related to increased risk of endometrial cancer, despite the known association between oestrogen replacement therapy and endometrial cancer. The results of some previous studies suggested an association with decreased risk of endometrial cancer (Bibbo et al, 1978; Hadjimichael et al, 1984), whereas others reported increased risks (Hoover et al, 1976; Autunes et al, 1979). We found no evidence that DES exposure was associated with increased risk of ovarian cancer. Previous studies suggested increased risk of ovarian (Hoover et al, 1977; Bibbo et al, 1978; Hadjimichael et al, 1984), and possibly cervical cancer (Bibbo et al, 1978; Hadmichael et al, 1984), but case numbers were small, and the findings were not statistically precise.

In summary, women who took DES while they were pregnant experience a 20–30% increased rate of breast cancer for many years thereafter. This modest increase in risk does not appear to be greater or lesser depending upon family history of breast cancer. It also appears not to be exacerbated by use of oral contraceptives or HRT. DES appeared not to increase the risk of other cancers, including endometrial or ovarian cancer.

### ACKNOWLEDGEMENTS

This project has been funded with Federal funds from the National Cancer Institute, National Institutes of Health, under Contract Numbers RFP NCI 33011-21 and CP50531-21.

### REFERENCES

Autunes CMF, Stolley PD, Rosenshein NB, Davies JL, Tonascia JA, Brown C, Burnett L, Rutledge A, Pokempner M and Garcia R (1979) Endometrial cancer and estrogen use. Report of a large case-control study. *New Engl J Med* **300**: 9–13

Beral V and Colwell L (1980) Randomised trial of high doses of stilboestrol and ethisterone in pregnancy: long term follow-up of mothers *B M J* **281**: 1098–1101

Bibbo M, Haenszel WM, Wied GL, Hubby M and Herbst AL (1978) A twenty-five-year follow-up study of women exposed to diethylstilbestrol during pregnancy. *New Engl J Med* **298**: 763–776

Breslow NE and Day NE (1987) Statistical Methods in Cancer Research II: The Design and Analysis of Cohort Studies. IARC Scientific Publication 94-2789. International Agency for Research on Cancer: Lyon, France

Calle EE, Mervis CA, Thun MJ, Rodriguez C, Wingo PA and Heath CW Jr (1996) Diethylstilbestrol and risk of fatal breast cancer in a prospective cohort of US women. *Am J Epidemiol* **144**: 645–652

CGHFBC (Collaborative Group on Hormonal Factors in Breast Cancer) (1997) Breast cancer and hormone replacement therapy: collaborative reanalysis of data from 51 epidemiological studies of 52 705 women with breast cancer and 108 411 women without breast cancer. *Lancet* **350**: 1047–1059

Clark LG and Portier KM (1979) Diethylstilbestrol and the risk of cancer. *New Engl J Med* **300**: 263–264

Colditz GA, Hankinson SE, Hunter DJ, Willett WC, Manson JE, Stampfer MJ, Hennekens C, Rosner B and Speizer FE (1995) The use of estrogens and progestins and the risk of breast cancer in postmenopausal women. *New Engl J Med* **332**: 1589–1593

Colton T, Greenberg ER, Noller K, Resseguie L, Van Bennekom C, Heeren T and Zhang Y (1993) Breast cancer in mothers prescribed diethylstilbestrol in pregnancy. *J Am Med Assoc* **269**: 2096–2100

Dieckmann WJ, Davis ME, Rynkiewicz LM and Pottinger RE (1953) Does the administration of diethylstilbestrol during pregnancy have therapeutic value? *Am J Obstet Gynecol* **66**: 1062–1081

Greenberg ER, Barnes AB, Resseguie L, Barrett JA, Burnside S, Lanza LL, Neff RK, Stevens M, Young RH and Colton T (1984) Breast cancer in mothers given diethylstilbestrol in pregnancy *New Engl J Med* **311**: 1393–1398

Hadjimichael OC, Meigs JW, Falcier FW, Thompson WD and Flannery JT (1984) Cancer risk among women exposed to exogenous estrogens during pregnancy *J Natl Cancer Inst* **73**: 831–834

Herbst AL and Scully RE (1971) Adenocarcinoma of the vagina. Association of maternal stilboestrol therapy with tumor appearance in young women *New Engl J Med* **284**: 878–81

Heston JF, Kelley JAB, Meigs JW and Flannery JT (1986) *45 years of cancer incidence in Connecticut: 1935–1979*. NCI Monograph Series no. 70, NIH Publication no. 86-2652. NCI: Bethesda, MD

Hoover R, Fraumeni JF, Jr, Everson R and Myers MH (1976) Cancer of the uterine corpus after hormonal treatment for breast cancer *Lancet* **XX**: 885–887

Hoover R, Gray LA and Fraumeni JF Jr. (1977) Stilboestrol (Diethylstilbestrol) and the risk of ovarian cancer *Lancet* 533–534

Hubby MM, Haenszel WM and Herbst AL (1981). Effects on the mother following exposure to diethylstilbestrol during pregnancy. In: *Developmental Effects of Diethylstilbestrol (DES) in Pregnancy*, Herbst AL, Bern HA (eds) pp 120–128. Thieme-Stratton Inc.: New York

Kelsey JL, Thompson WD and Evans AS (1986) *Methods in Observational Epidemiology*. Oxford University Press: New York

Newbold RR (1993) Gender-related behavior in women exposed prenatally to diethylstilbestrol. *Env Health Perspect* **101**: 208–213

Noller KL and Fish CR (1974) Diethylstylbestrol usage: its interesting past, important present, and questionable future. *Med Clin North Am* **58**: 793–810

 *© 2001 Cancer Research Campaign*

Ries LAG, Miller BA, Hankey BF, Kosary CL, Harras A and Edward BK, eds (1999) *SEER Cancer Statistics Review, 1973–1996*: Tables and Graphs. NIH Publication 99-2789. National Cancer Institute: Bethesda, MD

Schairer C, Lubin J, Troisi R, Sturgeon S, Brinton L and Hoover R (2000) Menopausal estrogen and estrogen-progestin replacement therapy and breast cancer risk *J Am Med Assoc* **83**: 485–491

Vessey MP, Fairweather DV, Norman-Smith B and Buckley J (1983) A randomized double-blind controlled trial of the value of stilboestrol therapy in pregnancy: long-term follow-up of mothers and their offspring *Br J Obstet Gynecol* **90**: 1007–1017

*© 2001 Cancer Research Campaign*
